# Vitamin C fortification: need and recent trends in encapsulation technologies

**DOI:** 10.3389/fnut.2023.1229243

**Published:** 2023-09-07

**Authors:** Vaibhav Kumar Maurya, Amita Shakya, David Julian McClements, Ramachandran Srinivasan, Khalid Bashir, Thiyagarajan Ramesh, Jintae Lee, Ezhaveni Sathiyamoorthi

**Affiliations:** ^1^Field Application Specialist, PerkinElmer, New Delhi, India; ^2^National Institute of Food Technology Entrepreneurship and Management, Sonipat, Haryana, India; ^3^Amity Institute of Biotechnology, Amity University Chhattisgarh, Raipur, India; ^4^Department of Food Science, University of Massachusetts Amherst, Amherst, MA, United States; ^5^Department of Food Science & Bioengineering, Zhejiang Gongshang University, Hangzhou, Zhejiang, China; ^6^Centre for Ocean Research (DST-FIST Sponsored Centre), MoES-Earth Science and Technology Cell (Marine Biotechnological Studies), Sathyabama Research Park, Sathyabama Institute of Science and Technology, Chennai, Tamil Nadu, India; ^7^Department of Food Technology, Jamia Hamdard University, New Delhi, India; ^8^Department of Basic Medical Sciences, College of Medicine, Prince Sattam Bin Abdulaziz University, Al-Kharj, Saudi Arabia; ^9^School of Chemical Engineering, Yeungnam University, Gyeongsan, Republic of Korea

**Keywords:** vitamin C, antioxidant, stability, fortification, encapsulation technique, delivery system

## Abstract

The multifaceted role of vitamin C in human health intrudes several biochemical functions that are but not limited to antioxidant activity, homoeostasis, amino acid synthesis, collagen synthesis, osteogenesis, neurotransmitter production and several yet to be explored functions. In absence of an innate biosynthetic pathway, humans are obligated to attain vitamin C from dietary sources to maintain its optimal serum level (28 μmol/L). However, a significant amount of naturally occurring vitamin C may deteriorate due to food processing, storage and distribution before reaching to the human gastrointestinal tract, thus limiting or mitigating its disease combating activity. Literature acknowledges the growing prevalence of vitamin C deficiency across the globe irrespective of geographic, economic and population variations. Several tools have been tested to address vitamin C deficiency, which are primarily diet diversification, biofortification, supplementation and food fortification. These strategies inherit their own advantages and limitations. Opportunely, nanotechnology promises an array of delivery systems providing encapsulation, protection and delivery of susceptible compounds against environmental factors. Lack of clear understanding of the suitability of the delivery system for vitamin C encapsulation and fortification; growing prevalence of its deficiency, it is a need of the hour to develop and design vitamin C fortified food ensuring homogeneous distribution, improved stability and enhanced bioavailability. This article is intended to review the importance of vitamin C in human health, its recommended daily allowance, its dietary sources, factors donating to its stability and degradation. The emphasis also given to review the strategies adopted to address vitamin c deficiency, delivery systems adopted for vitamin C encapsulation and fortification.

## 1. Introduction

**Figure 1:**
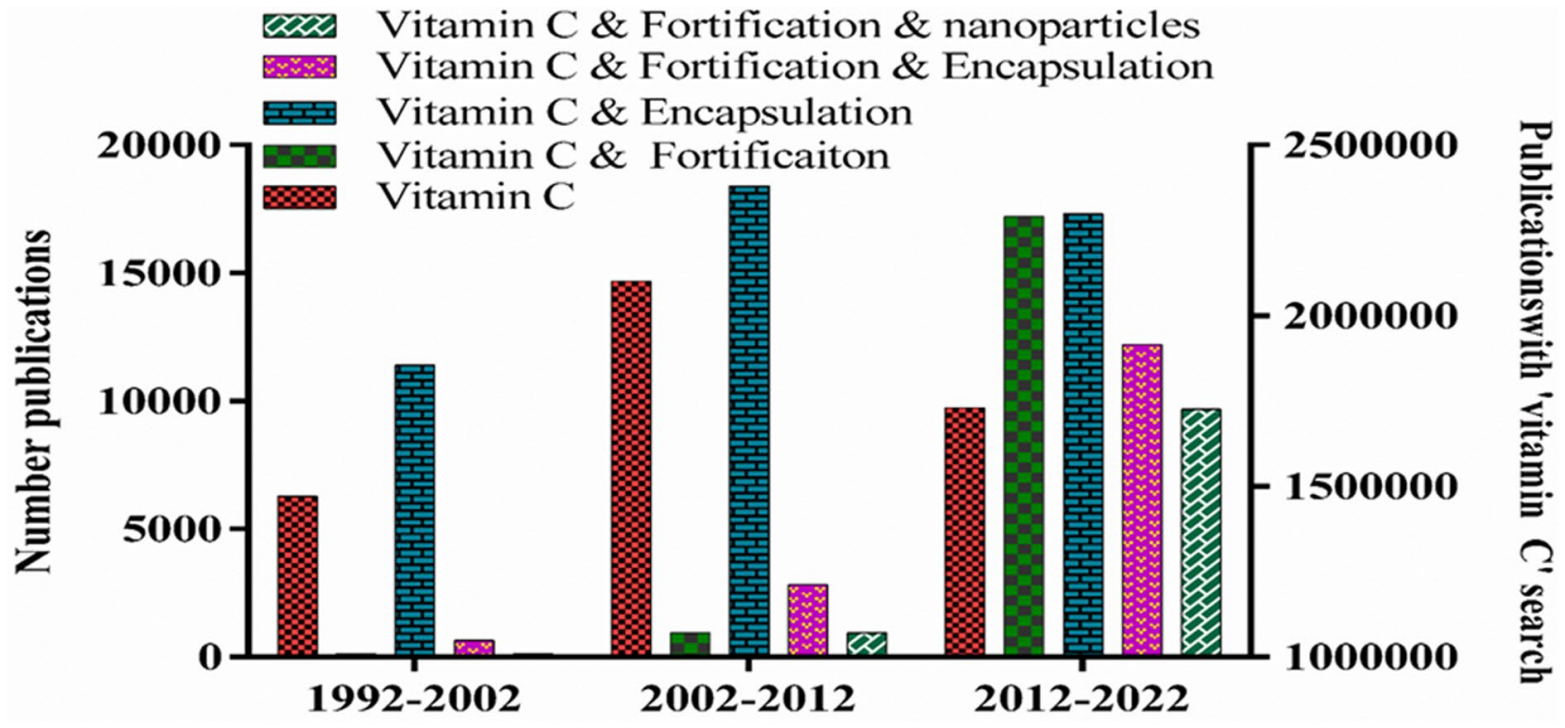
Number of publications with keywords “vitamin C”, “vitamin C and fortification” or “vitamin C and fortification and encapsulation” and “vitamin C and fortification and nanoparticles.”

Vitamin C (ascorbic acid) has been well documented for its antioxidant activity and other biological activities (1). Humans cannot synthesize this essential nutrient and so must obtain it from their diet (2, 3). Vitamin C deficiency has been linked to several diseases in humans (4, 5), most notably scurvy, which are related to its key role in numerous biochemical functions, including collagen synthesis, amino acid synthesis, blood pressure control, atherogenesis, homoeostasis, neurotransmitter production, and osteogenesis (5–7). In addition to its role as a vitamin, ascorbic acid has also been shown to exhibit various nutraceutical functions, including anticancer effects. There have been an increasing number of publications on vitamin C over the past few decades, with many of them focusing on food fortification (Figure 1). The World Health Organization (WHO) states that food fortification is one of the most effective, safe, and economical ways of addressing nutrient deficiencies (8). However, the inclusion of vitamin C in functional foods and beverages is often challenging due to its chemical instability and low bioavailability (9–12).

Consequently, many researchers have focused on developing strategies to overcome these challenges (13–17). This review will begin by discussing the physicochemical attributes, biosynthesis, food sources, recommended dietary allowance, and available fortification strategies for vitamin C. Then the challenges associated with food fortification are discussed, and the important role of colloidal delivery systems in encapsulating, protecting, and delivering vitamin C is highlighted. The knowledge presented in this article may facilitate the development of more efficacious strategies for fortifying foods and beverages with this important micronutrient.

### 1.1. History

The connection between vitamin C deficiency and scurvy can be traced back to 1700 BC when the Ebers Papyrus defined the characteristic features of scurvy. There is also evidence of the role of food deficiencies in the writings of several ancient scholars, including Hippocrates in Greece (460 BC), Susrutrain India (400 BC), and Chang Chi in China (200AD). From the sixteenth to eighteenth centuries, there was a growing understanding that dietary deficiencies were causing diseases in sailors (18). Eventually, this led to the first scientific publication on the subject, “A Treatise of Scurvy”, by James Lind in 1753, where he emphasizes the importance of lemons, oranges, and fresh green vegetables in the prevention of scurvy (19). Almost, two centuries later, Albert Szent-Gyorgyi published his observations on the extraction of vitamin C (a “sugar-like crystal”) in the Biochemical Journal under the title “Observation on the function of peroxidase systems and the chemistry of the adrenal cortex: Description of a new carbohydrate derivate” (20). Later, W. M. Haworth elucidated the structure of these sugar-like crystals and named it hexuronic acid. Then, King and Waugh (21) extracted these sugar-like crystals from lemon juice and named it ascorbic acid, which was described in their article “The chemical nature of vitamin C” (21). Albert Szent-Gyorgyi was eventually granted the Nobel prize in Physiology or Medicine for his research on vitamin C (22).

### 1.2. Molecular and physicochemical properties

**Figure 2:**
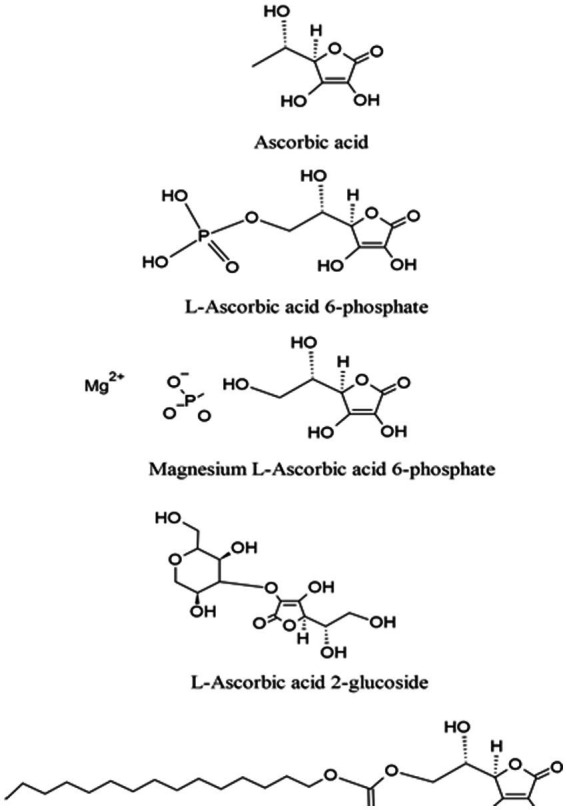
Vitamin C and its analogs.

Vitamin C is a low molecular weight carbohydrate with an enediol structure (Figure 2), which makes it a natural electron donor. The enediol structure also makes it susceptible to chemical degradation when exposed to changes in environmental conditions, such as pH, temperature, humidity, salt, and radiation (23). Several vitamin C analogs with differing physicochemical characteristics have also been synthesized (Figure 2). Researchers have classified these vitamin C analogs depending on their water-solubility and ability to raise vitamin C serum levels. Based on their physicochemical characteristics, these analogs can be categorized into:

- Hydrophilic ascorbic acid: This group includes l-ascorbic acid 2-glucoside, magnesium l-ascorbic acid 6-phosphate, and l-Ascorbic acid 6-phosphate
- Hydrophobic ascorbic acid: This group includes tetra-isopalmitoyl ascorbic acid and l-ascorbyl 6-palmitate (10).

Based on their ability to increase blood serum levels of vitamin C they can also be categorized into two groups according to their potency (10):

- *Strong potency*: This group includes l-ascorbate 2-phosphate, 6-bromo-6-deoxy-l-ascorbic acid, l-ascorbate 2-triphosphate, and ascorbic acid 2-*O*-α-glucoside;
- *Weak potency*: This group includes l-ascorbate-*O*-methyl ether, l-ascorbyl-2-sulfate, and l-ascorbyl palmitate.

### 1.3. Biosynthesis

Some animal species and green plants can naturally synthesize vitamin C through the innate glucuronic acid biochemical pathway (24). During evolution, however, humans appear to have lost a key enzyme (l-gulono-1,4 lactone oxidase) within the ascorbic acid biosynthesis pathway (25–27). Consequently, they must obtain this vitamin from their diet.

Dietary vitamin C is usually present in two different forms: ascorbic acid (reduced form) and dehydroascorbic acid (oxidized form). Initially, researchers speculated that vitamin C was only absorbed by the human body through passive diffusion due to its highly hydrophilic nature. Later, however, researchers identified a sodium-dependent vitamin C transporter responsible for the absorption of ascorbic acid (28). While other researchers found that the absorption of dehydroascorbic acid was mainly through glucose transporter isoforms GLUT1 and GLUT3 (29).

Researchers also observed a shift in the absorption mode depending on the vitamin C levels in dietary sources. For instance, at higher concentrations the uptake of vitamin C occurs mainly by passive diffusion while at lower concentrations it mainly follows carrier-mediated active transport (30). However, the precise threshold at which this transition occurs is still a matter of investigation. The absorption efficiency of vitamin C within the human gastrointestinal tract (GIT) is also dose-dependent, i.e., at lower vitamin C doses (<180 mg/day) it has been reported to be up to 80–90% but at higher doses it is reported to be considerably lower (30).

## 2. Dietary sources

**Figure 3:**
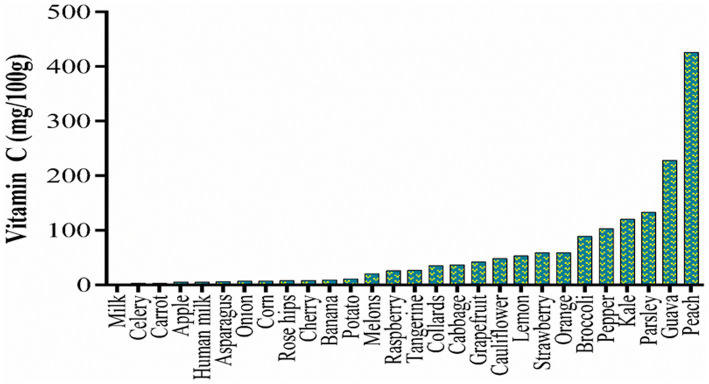
Vitamin content in different food items.

Due to the lack of a vitamin C biosynthetic pathway, humans rely on dietary sources to maintain an optimal vitamin C serum level. Fruits and vegetables are the major sources of vitamin C in the human diet (around 90%), with the remainder coming from animal sources (4). Several studies have reported the vitamin C content of different food types (Figure 3) including milk (31, 32), apple (33), banana (34), cherry (35), grapes (36), guava (37), lemons (38), melon (39), orange (35), peach (40), raspberry (41), rosehip (42), strawberry (43), tangerine (44), asparagus (45), broccoli (46), cabbage (47), carrot (48), celery (49), collards (50), kale (45), onions (51), and pepper (45). Vitamin C may also be consumed in the form of dietary supplements, such as capsules or tablets.

## 3. Stability in foods

Vitamin C is a chemically active molecule that may undergo appreciable degradation in foods during processing, storage, and distribution (12). This degradation is mainly due to the hydrolytic opening of the lactone ring, thus resulting in the formation of a biologically inactive compound 2,3-deketogluconic acid (12). This degradation reaction is accelerated when the vitamin C is exposed to oxygen, transition metals, heat, and alkaline conditions (Table 1). For example, a significant proportion of vitamin C has been reported to be lost during the storage of potatoes, cabbages, and apples (64). The degree of loss depends on food matrix type and environmental conditions (stress). For example, boiling of potatoes was reported to cause a 40% loss in vitamin C content (2). Among the cooking methods steam seem to be most detrimental method for vitamin C. In contrast, some food processing operations stabilize vitamin C by inactivating enzymes (such as oxidases) that might promote its oxidation (2).

**Table 1:** Vitamin C retention in fortified foods during their storage.

Food categories	Food commodity	Storage conditions	Storage time (days)	Vitamin C retention	References
Milk	Fortified milk	25°C/3-layered packaging material	30	1	(12)
		25°C/6-layered packaging material	30	49	
		25°C/6-layered packaging material	120	25	
	Fortified milk	4°C	5	90.6	(52)
	Evaporated milk	23°C	365	75	(53)
Cereal based food	Bread	25°C, polyethylene bags	7	15	(54)
	Fiber fortified bread	25°C/moisture 45%	7	3	(55)
	Bread without fiber	25°/moisture 37%	7	14	
	Bread fortified with l-ascorbate 2-monophosphate and reduced Iron	25°C	6	52	(56)
	Bread fortified with ascorbic acid and reduced Iron	25°C	6	18	
	Ready-to-eat cereals	23°C	365	71	(53)
	Ready-to-eat cereals	Room temperature	360	60	(57)
	Cereals	40°C	90		(58)
		22°C	180		
	Bran flakes	25°C/7% moisture	30	95	(56)
		40°C/11% moisture	30	20	
Fruit beverages	Strawberry drink	4–6°C	90	67.7	(59)
	Yellow passion fruit juice	37°C	14	0	(60)
	Blood orange juice	4.5°C	49	25.1	(61)
	Powder fruit drinks	21°C	1	84	(62)
	Dry fruit drink mix	23°C	365	94	(53)
	Apple juice	23°C	365	68	(53)
	Cranberry juice	23°C	365	81	
	Grapefruit juice	23°C	365	81	
	Pineapple juice	23°C	365	78	
	Grape drink	23°C	365	76	
	Orange drink	23°C	365	80	
Vegetable beverages	Tomato juice	23°C	365	80	(53)
	Vegetable juice 68 0.44	23°C	365	68	(53)
Carbonated drinks	Carbonated beverages	23°C	365	60	(53)
Cola beverages	Cola drinks fortified with ascorbic acid	15°C	365	83.1	(53)
	Cola drinks fortified with L-ascorbate 2-monophosphate	15°C	365	97	
	Cola drinks fortified with L-ascorbate 2-polyphosphate	15°C	365	97.7	
	Cola drinks fortified with ascorbic acid	25°	365	70	
	Cola drinks fortified with L-ascorbate 2-monophosphate	25°	365	90	
	Cola drinks fortified with L-ascorbate 2-polyphosphate	25°	365	95.4	
	Cola drinks fortified with ascorbic acid	35°C	365	63.8	
	Cola drinks fortified with L-ascorbate 2-monophosphate	35°C	365	68.4	
	Cola drinks fortified with L-ascorbate 2-polyphosphate	35°C	365	93.8	
Coffee product	Cocoa powder	23°C	365	97	(53)
Fruit/vegetable flakes	Dried apple chips	7°C, RH 45%	270	80.4	(63)
		18°C, RH 90%	270	63.1	
	Potato flakes fortified with ascorbic acid	25°C	129	18	(64)
	Potato flakes fortified with L-ascorbate 2-monophosphate,	25°C	129	88	
	Potato flakes fortified with L-ascorbate 2-polyphosphate	25°C	129	84	

## 4. Vitamin C bioavailability

**Figure 4:**
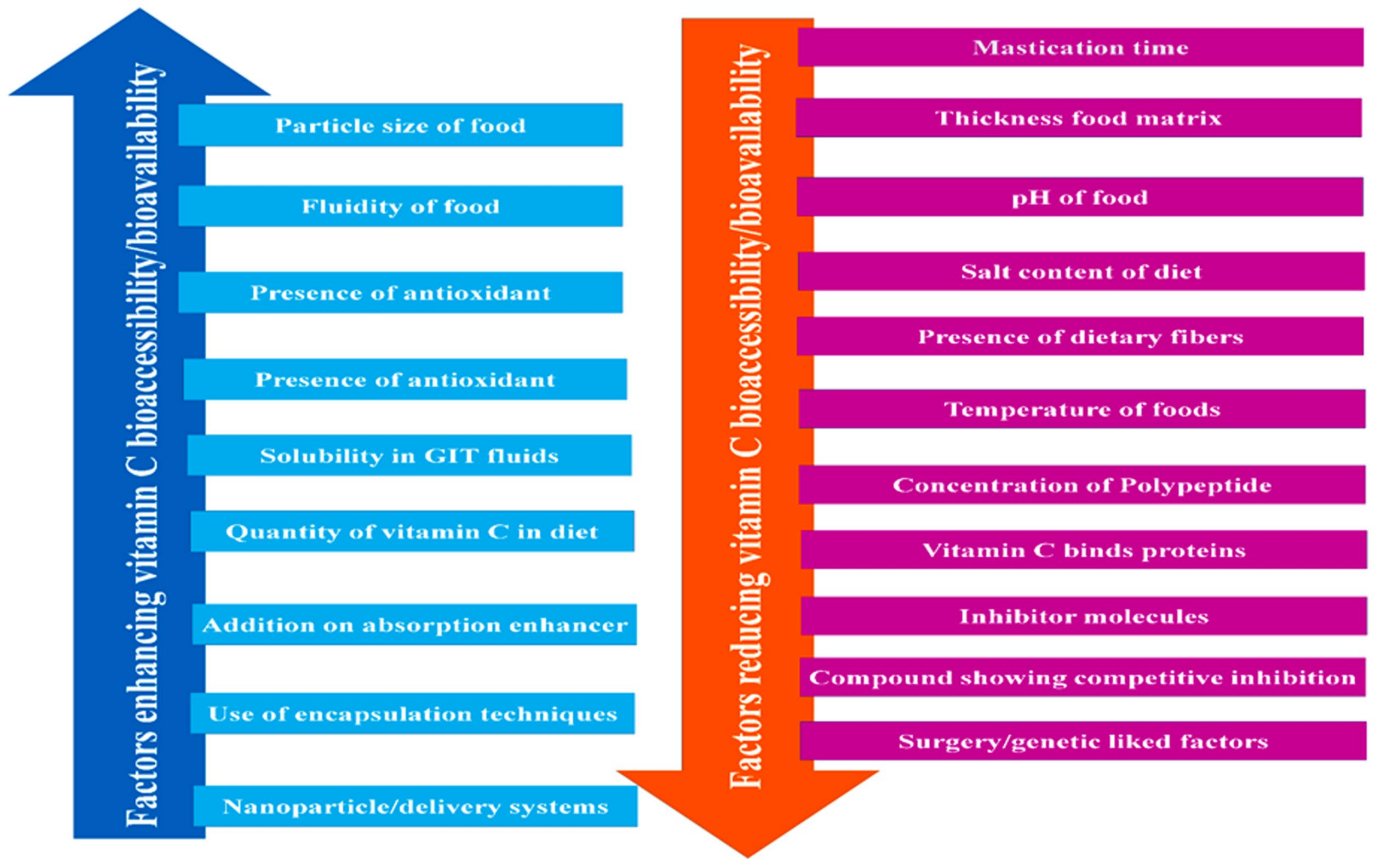
Factor influencing vitamin C bioaccessibility/bioavailability in GIT.

The biological efficacy of a vitamin depends on the quantity absorbed and utilized by the body rather than the amount consumed. The proportion of a vitamin absorbed in its active state is referred to as its bioavailability (2). The bioavailability of vitamin C depends on an array of factors, including the dose consumed, the composition and structure of the food matrix, the environmental conditions experienced during processing, storage, and distribution, and passage through the gastrointestinal tract (Figure 4 and Table 2). The bioavailability of vitamin C in foods is often considered to be equivalent to that of the purified form when the dose lies within the required nutritional range (15–200 mg) (2). However, it tends to fall by more than 50% when higher amounts (e.g., >1000 mg) are ingested (2). The conversion of ascorbic acid to dehydroascorbic acid in foods or the gastrointestinal tract can also reduce the bioactivity of vitamin C. A range of chemically synthesized ascorbic analogs have been developed to improve the chemical stability and bioavailability of vitamin C, including ascorbate 2-sulfate, ascorbate 2-monophosphate, and ascorbate 2-triphosphate (2).

**Table 2:** Bioaccessibility of vitamin C in different supplement and food matrices.

Food/supplements	Bioaccessibility	*In vitro* digestion applied	Major factor governing bioavailability	Reference
Dietary supplements	49–99%	Saliva Gastric Duodenal juice Bile juice	Encapsulation and other components	(65)
Infant formula	0.1–44%	Saliva Gastric Duodenal juice Bile juice	Encapsulation and other components	(65)
Fortified foods	0.3–1.4%	Saliva Gastric Duodenal juice *Bile juice*	Encapsulation and other components	(65)
Fruit juice	51–83%	Gastric Small intestine	pH of gastric and small intestine juice	(66)
Broccoli inflorescences	93%	Gastric Small intestine	pH of gastric and small intestine juice	(67)
Pomegranate juice	71%	Gastric Small intestine	pH of gastric and small intestine juice	(68)
Fruit juice-soymilk	20.5–23.2%	Gastric Small intestine	Vitamin C binding protein, other vitamins and metal ions	(69)
milk	10.9–13.1%	Gastric Small intestine	Vitamin C binding protein, other vitamins and metal ions	(69)
water	(11.1–14.2%)	Gastric Small intestine	Vitamin C binding protein, other vitamins and metal ions	(69)
Fruit beverages	16.3–56.0%	Gastric Small intestine	Milk protein	(70)
Fruit beverages-whole milk	70.17%	Gastric Small intestine	Emulsification by milk addition	(71)
Fruit beverages -skim milk	62.41%	Gastric Small intestine	Emulsification by milk addition	(71)
Fruit beverages-soy milk	12.58%	Gastric Small intestine	Emulsification by milk addition	(71)
Orange segments	54%	Gastric Small intestine	NA	(72)
homogenized orange segments	38%	Gastric Small intestine	Homogenization	(72)

## 5. Vitamin C deficiency

### 5.1. Indicators for vitamin C deficiency

The vitamin C status of a person or population may be established by diet-based assessments or analytical measurements. Diet-based assessments rely on analysis of food consumption patterns and frequencies. In this approach, subjects typically complete questionnaires related to their daily food consumption and then their vitamin C intake can be calculated from food databases. Analytical methods rely on measurement of the vitamin C levels in the serum of individuals, which can be achieved using various analytical methods including liquid chromatography and mass spectrometry. Plasma/serum vitamin C level is recognized as a biomarker for vitamin C status: <11 μmol/L (deficient), ≥11–28 μmol/L (suboptimal), and >28 μmol/L (sufficient) (4).

### 5.2. Vitamin C deficiency across the globe

**Figure 5:**
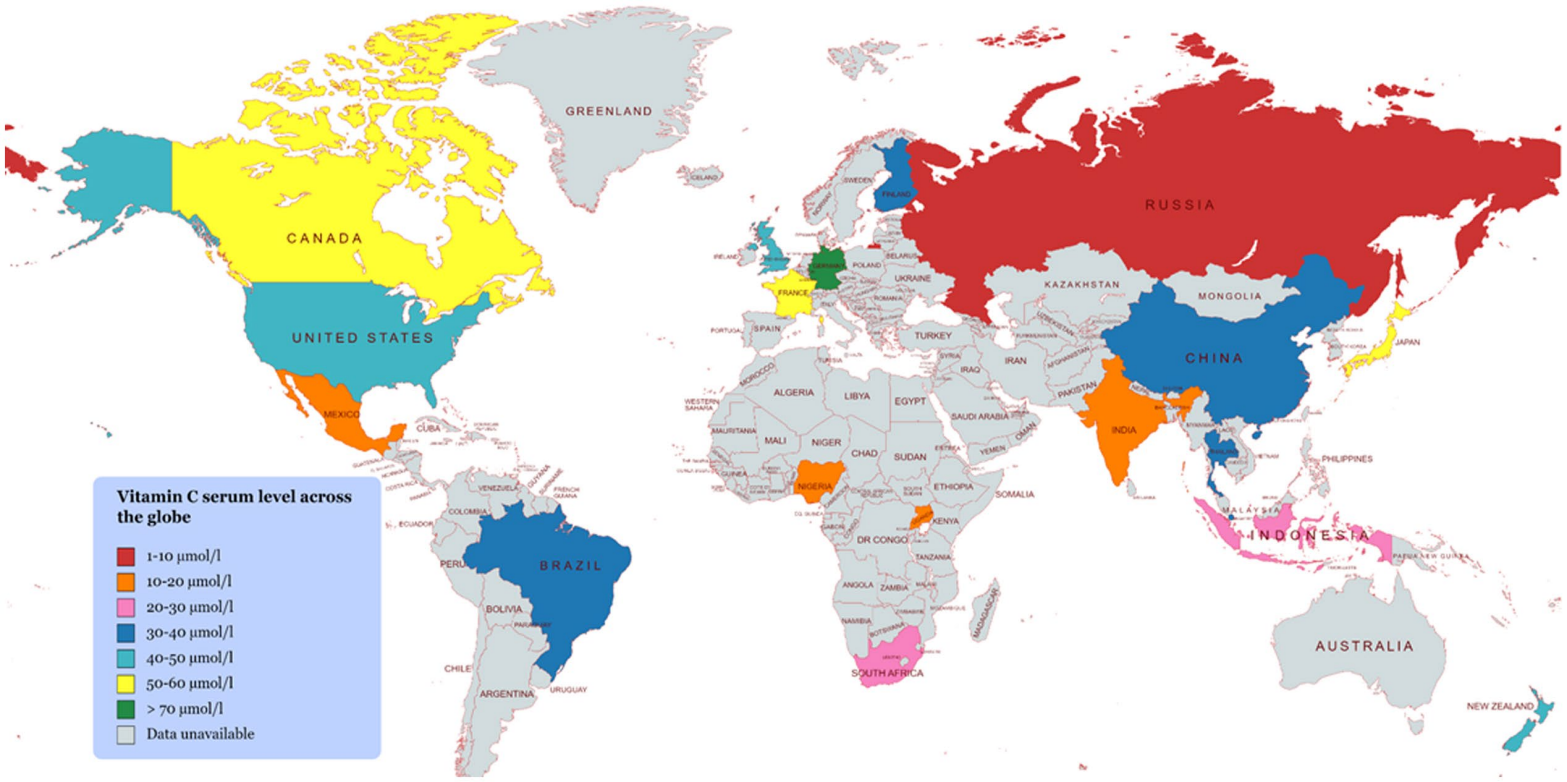
Vitamin C serum level across the globe.

Despite improvements in the human diet over the past century, there are still high levels of vitamin C deficiency in some populations around the world (Figure 5) (73–76). For example, the EPIC-Norfolk survey conducted a vitamin C assessment of a relatively large sample size (22,400 participants) and observed a higher vitamin C deficiency in males than females (77, 78). This difference in vitamin deficiency status between male and female population is mainly governed by life style (smoking), low consumption of vitamin C supplements. Researchers have also reported significant differences in vitamin C status for different populations e.g., a lower level of vitamin C deficiency in the overall British population (14%) than in the Scottish one (20%) (79, 80). European and American populations also significantly vary in their vitamin C status (80). The prevalence of vitamin C deficiency is more pronounced in some other countries. For instance, its prevalence was widespread (up to 60%) in the female population in Quinto, Ecuador and similar observations have been made in other South American and African populations (81–89). In Asia, vitamin C status also significantly varied between and within countries. For example, low vitamin C status was recorded in the Indian population as compared to the Chinese population, with this difference being more prominent in the female population (90–92). This discrepancy in vitamin C status among different populations is mainly attributed to differences in the types of foods that are available and commonly consumed (4).

### 5.3. Health concerns related to vitamin C deficiency

Various factors contribute to the vitamin C requirements and status of individuals, including geographical, demographic, diet, socioeconomic environmental, and health factors (4, 93) (Table 3). Due to its many roles in human health; suboptimal vitamin C levels lead to a variety of undesirable health effects, including oxidative stress, malfunction of key biochemical pathways, and inhibition of the synthesis of key biological components, which can lead to diseases, such as scurvy. Moreover, studies have highlighted the important role of vitamin C in the prevention of cardiometabolic disorders, diabetes, and cancer (144, 145). Vitamin C also plays role in hormone regulation, neurotransmitter production, immunological functions, connective tissue development, and several other important biological functions (2, 6, 146, 147).

**Table 3:** Factors affecting vitamin C status in human.

Class of factors	Factors	Major contributors	Inference	References
Diet dependent factors	Dose intake	Fruit and vegetables	Vitamin C status depends on ingested amount Sugar and fat decrease the vitamin C intake	(80–82, 92, 94–99)
	Staple foods	Cereals and starchy tubers rains	Stable food-based diet decreases the vitamin C intake as they are poor in vitamin C content	(100, 101)
	Cooking practices	Washing, drying, boiling and steaming	Washing allow leaching of vitamin C Prolong drying, heating and steaming cause vitamin C degradation	(102–106)
	Supplement consumed	High dose containing formulation	Users have improved vitamin C status than deficient	(78, 96, 97, 99, 107, 108)
Socioeconomic factor	Socioeconomic status	Cost of food	Population with low economic status are note able to afford vitamin rich food items	(78, 79, 82, 89, 92, 97, 99, 109–112)
	Education and social class	Awareness	Population with lower education and manual occupation are deficient in vitamin C	(78, 80, 96, 98, 110)
	Institutionalization	Low dietary intake	Institutionalized population (prisoners, priests and boarding school children) are vitamin C deficiency due to low intake of vitamin C rich food	(79, 113)
Environmental factors	Geography	Altitude and latitude	Consumption pattern depends on local food which varies with geography	(79, 89, 92, 97, 98, 114–116)
	Season	Variation in agricultural crops	Seasonal variation has major impact of type of crops as well as their vitamin C content	(90–92, 108)
	Climate	Extreme weather	Draught and frost cause damage of crops thus limiting food diversity	(117)
	Pollution	Smoke and particulate maters	Population cause depletion of vitamin C and cause oxidative stress	(118–121)
Demography	Gender	Fat vs body weight ratio	Male has low vitamin C status than female due to low The difference becomes less prominent in some low- and middle-income population	(78, 81, 91, 92, 96–99, 103, 107, 108, 110, 111, 114, 116, 122–124)
	Age	Diet preference	Children and elderly person have high vitamin C status due to low fat: body weight ratio	(78–80, 92, 96, 99, 107, 114, 125, 126)
	Race	Genetic variation	South Asian people and African-Caribbean has low vitamin C status that Caucasians Indians have low vitamin C status than Chinese Disparity could be due to variation in consumption pattern Difference in vitamin C status become more significant in female populations	(99, 103, 108, 127)
Host health dependent factors	Bodyweight/ BMI	Fat vs body weight ratio	Individuals having higher body weight/ BMI are lower in vitamin C status	(83, 92, 95, 96, 99, 107, 108, 111, 128, 129)
	Physical activity	Consumption pattern	Person having high physical activity consumed nutrient rich food hence have high vitamin C status	(78, 107)
	Pregnancy and lactation	Vitamin C transfer to fetus and hemodilution	Pregnancy lowers vitamin C status	(86, 130)
	Genetic variants	Polymorphisms in alleles linked to vitamin C transported (SVCT1) and haptoglobin (Hp2-2)	Vitamin C status lowers under high oxidative stress	(112, 131–135)
	Smoking habits	oxidative stress.	Smokers have high vitamin C deficiency	(78, 80, 91, 92, 95–97, 99, 107, 108, 111, 112, 114, 123, 125, 136–139)
	Disease status	Oxidative stress and inflammation	Host suffering from communicable and noncommunicable diseases has low vitamin C status	(140–143)

### 5.4. Recommended dietary allowance

The daily requirement of vitamin C depends somewhat on the gender, age, health status, and lifestyle of individuals (Table 4). However, the Institute of Medicine advises 75 mg/day (female) and 90 mg/day (adult men) as the recommended dietary allowance (RDA) (146, 148–152). The RDA is the amount of vitamin C that should be consumed daily to maintain good health [Dietary Reference Intakes: Thiamin R and Choline (153); IOM and FNB (128, 154)]. The Institute of Medicine also recommends the intake of35 mg of additional vitamin C for habitual smokers over the general population (2, 155).

**Table 4:** Recommended dietary allowance for vitamin C.

Age group (year)	Recommended dietary allowances			Tolerable upper intake	
	Female	Male	Physical status (Pregnancy/lactation)	Female	Male
0–0.6	40	40	NA	NA	NA
<1	50	50	NA	NA	NA
1-3	15	15	NA	400	400
4-8	25	25	NA	650	650
9-13	45	45	NA	1200	1200
14-18	65	75	80/115	1800	1800
19+	75	90	85/120	2000	2000
Habitual smokers	35 mg additional				

### 5.5. Vitamin C intake and the current supply

**Figure 6:**
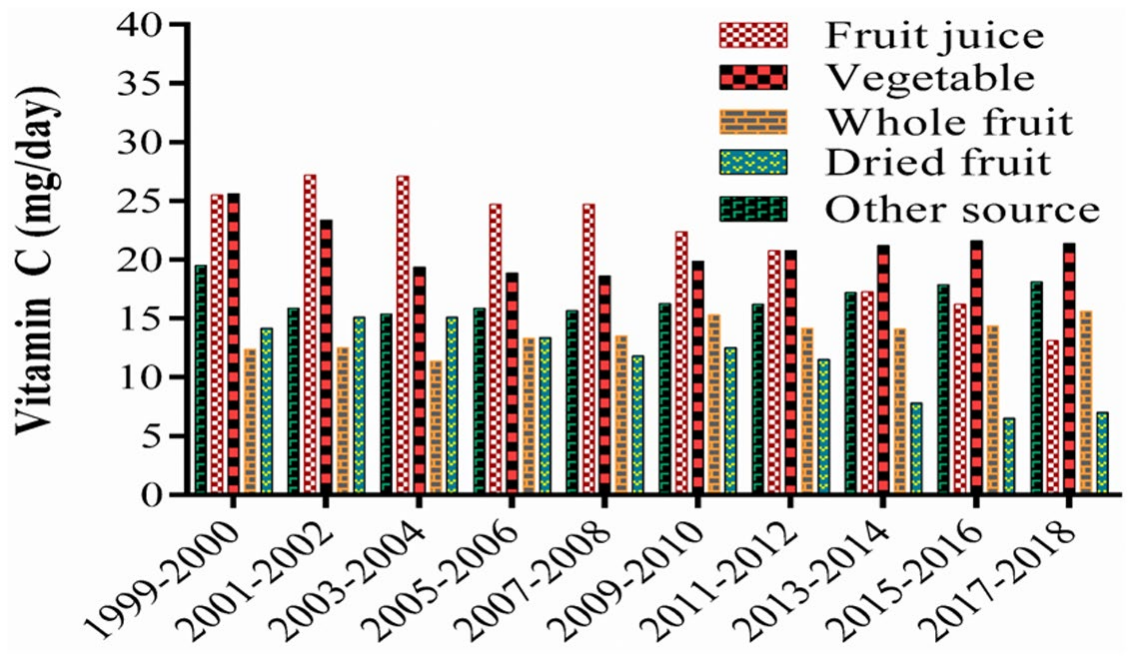
Major contributor of vitamin C in human diet.

Food consumption patterns vary according to geography, demography, socioeconomic status, and dietary preferences. Researchers have reported that a significant proportion of vitamin C in the diet comes from fruit juices, vegetables, whole fruits, and dried fruits (Figure 6) and that vitamin C consumption has declined between 1999 and 2018 (148). In many populations, the current supply of vitamin C is sufficient to meet the RDA but, in some populations, this is not the case. As a result, strategies need to be developed to address potential vitamin C deficiencies in these populations.

## 6. Strategies adopted to address vitamin C deficiency

In general, there are several approaches to addressing vitamin deficiency: (i) diet; (ii) biofortification; (iii) food fortification; and (iv) dietary supplements (15–17). In this section, several of these approaches are discussed in the context of vitamin C.

### 6.1. Diet

Diet-based approaches involve the addition of vitamin C-rich food items to the diet, such as lemons, oranges, kiwi, and other fruits and vegetables (156–158). However, this strategy depends on the affordability and availability of these items, as well as typical eating patterns in the target populations (10, 15, 17). The inclusion of vitamin C-rich food items that have a high bioavailability is crucial for the success of this approach (10). Educating the target population about health concerns linked with vitamin C deficiency, as well as the sources of affordable vitamin C-rich foods are also important.

### 6.2. Supplementation

Dietary supplements are also a successful means of ensuring that people have sufficient levels of vitamins and minerals in their diet to prevent health problems (159, 160). Supplements are typically available as capsules, tablets, or powders containing vitamin C alone or in combination with other nutrients (161). These formulations are designed to contain levels of vitamin C or its analogs that help ensure people meet the RDA. They must be carefully formulated to ensure the vitamins remain stable during storage and after ingestion. The major limitation of supplementation is that they are not affordable or desirable for many people.

### 6.3. Biofortification

Biofortification approaches rely on increasing the micronutrient levels in agricultural staple crops using selective breeding, crop management, and/or genetic engineering approaches. Researchers have already used these approaches to increase the vitamin C levels in various kinds of fruits and vegetables, including strawberries, tomatoes, and potatoes (Table 5). For instance, researchers overexpressed the GDP-l-galactose phosphorylase (GGPor VTC2) gene in transgenic tomatoes to enhance their vitamin C content by 3- to 6-fold (163). Similarly, genes have been inserted into corn to increase its vitamin C content appreciably (165). Another study reported a 2-fold increase in the overexpression of the GDP-l-galactose phosphorylase gene in strawberries, leading to an increase in their vitamin C level (163).

**Table 5:** Transgenic biofortified pants with improved vitamin C content.

Fortified crop/plants	Gene used	Target gene/pathway	Process	Vitamin C content	Reference
Strawberry	GDP-l-galactose phosphorylase	NADPH-dependent D-galacturonate reductase	Overexpression	2-fold increase	(162)
	GDP-l-galactose phosphorylase	l-galactose pathway gene	Overexpression	2-fold increase	(163)
Tomato	GDP-l-galactose phosphorylase	Smirnoff-wheeler pathway	Overexpression	3–6-fold increase	(163)
Potato	GDP-l-galactose phosphorylase	Smirnoff-wheeler pathway	Overexpression	3-fold increase	(163)
	l-gulono-γ-lactone oxidase	Dehydroascorbate reductase		141% increase	(164)
Corn	Dehydroascorbate reductase (dhar)	Smirnoff-wheeler pathway	Overexpression	6-fold increase	(165)
	Dehydroascorbate reductase (dhar)	Smirnoff-wheeler pathway	Overexpression	2–4-fold increase	(166)
lettuce	Ggulono lactone oxidase	L-Ascrobic acid pathway	Overexpression	7-fold increase	(167)
Tobacco	GDP-mannose pyrophosphorylase	Smirnoff-wheeler pathway	Overexpression	2-folds increase	(168)
*Arabidopsis*	GDP-galactose phosphorylase	Smirnoff-wheeler pathway	Overexpression	7-folds increase	(169)

### 6.4. Food fortification

Food fortification is an effective, safe, and affordable approach to meeting the nutritional requirements of certain populations (170). The efficacy of vitamin fortification is enhanced when it can be integrated into an existing food supply network (15, 17). However, it is important to select appropriate food types for fortification with vitamin C. Knowledge of the vitamin C requirements and status of the target population is required. Information about the dietary patterns of this population is also required to establish the most common types and amounts of foods and beverages consumed. An understanding of the physicochemical properties of vitamin C and its analogs is also required, such as its stability, solubility, and interaction characteristics. Then, effective methods of incorporating vitamin C into these products in a stable and bioavailable form, without adversely impacting their organoleptic attributes or affordability, are required (171, 172). Vitamin C is a water-soluble molecule that can often be simply dissolved into aqueous solutions and food matrices. However, it may physically interact with other components or chemically degrade, which can reduce its efficacy or decrease food quality attributes. Consequently, fortification must be carried out carefully.

**Figure 7:**
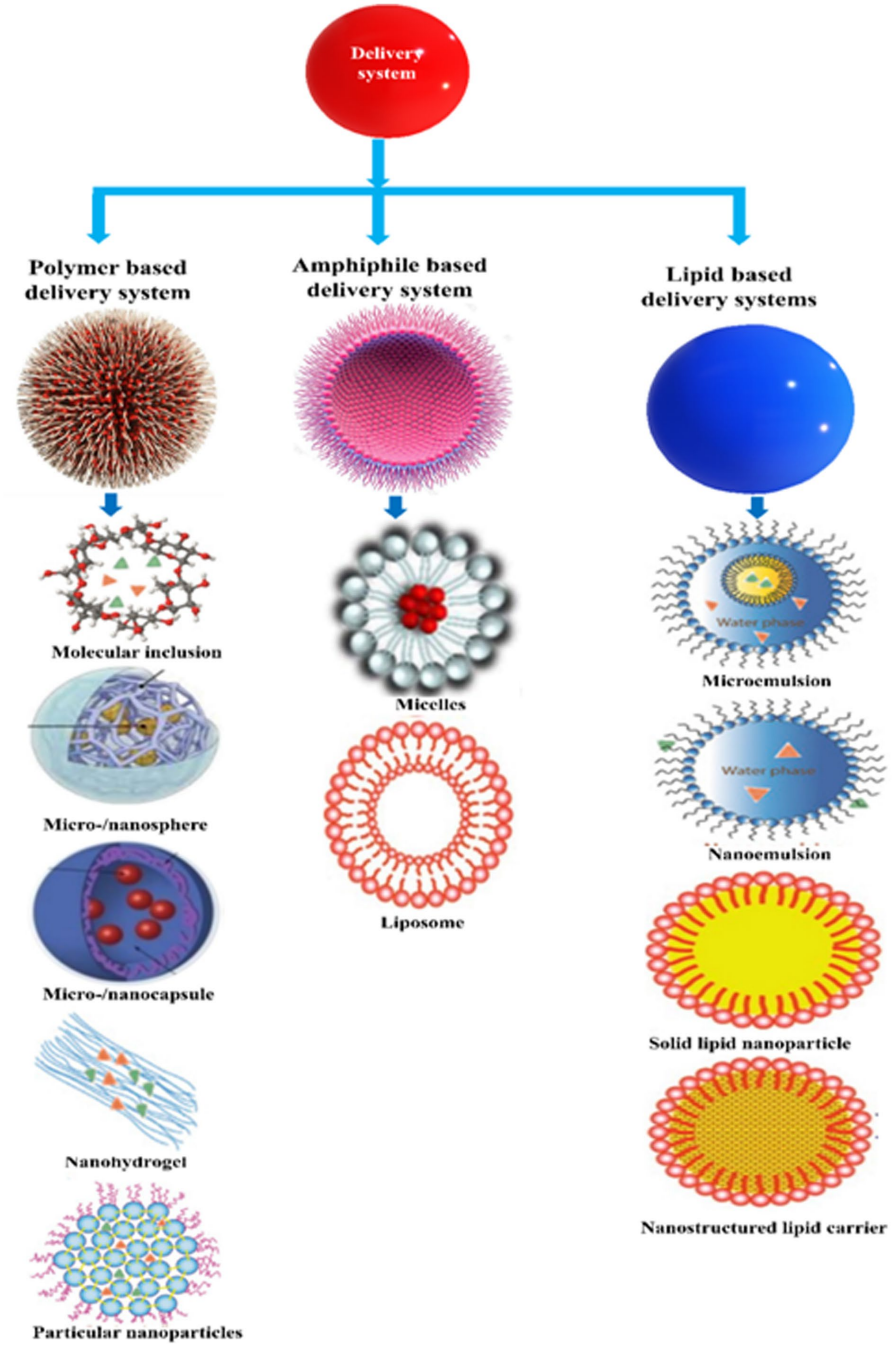
Delivery system adopted for vitamin C encapsulation.

Various food matrices have already been fortified with vitamin C, however, significant losses can occur during storage, processing, and distribution (Tables 1, 6). The extent of these losses depends on food matrix effects and the environmental conditions the foods are exposed to (189–207). For instance, thermal processing and trace metals can promote rapid degradation of vitamin C (40, 46, 104, 106), thereby reducing its potentially beneficial health effects. These challenges can often be overcome using suitable encapsulation techniques (Figure 7 and Supplementary Table S1).

**Table 6:** Vitamin C fortified products and its challenges.

Food matrix	Vitamin C fortificant	Enhanced functionalities of fortified food	Major limiting factors	Reference
Liquor chocolate	Dehydrogenated ascorbic acid	Improved antioxidant properties	Poor stability Sour taste	(173)
Sausage	l-ascorbic acid	Improved antioxidant properties	Poor stability Sour taste	(174)
Milk	l-ascorbic acid	Improved antioxidant properties	Poor stability Sour taste	(175)
Edible coating	l-ascorbic acid	Improved antioxidant properties Antibacterial properties	Poor stability Sour taste	(176)
Mao fruit juice	l-ascorbic acid	Improved antioxidant properties High iron content	Poor stability Sour taste	(177)
Fortification formulation	l-ascorbic acid	Improved antioxidant properties	Poor stability	(178)
Meat patties	l-ascorbic acid	Improved antioxidant properties	Poor stability Sour taste	(179)
Fish feed	l-ascorbic acid sodium	Improved antioxidant properties Cost effective	Poor stability than ascorbic acid After baking Sodium ascorbate exhibits anti-nutritional effect on protein	(180)
Dry fermented sausages	l-ascorbic acid sodium	Improved antioxidant properties Cost effective	Poor stability than ascorbic acid	(181)
Black rice baking products	2-*o*-d-glucopyranosyl-l-ascorbic acid	Anti-oxidation	High cost Low yield	(182)
Beef patties	2-*O*-d-glucopyranosyl-l-ascorbic acid	Anti-oxidation for fat High stability	High cost Low yield	(183)
Patent formulation for food fortification	2-*O*-d-glucopyranosyl-l-ascorbic acid	Improved antioxidant properties	High cost Low yield	(184)
Maize starch	l-ascorbic acid palmitic acid ester	Improved antioxidant properties	Poor thermal stability Low chemically stability	(185)
Bakery product	l-ascorbic acid palmitic acid ester	Improved antioxidant properties High heme iron and calcium content	Poor thermal stability Low chemically stability	(186)
Milk formula	l-ascorbic acid palmitic acid ester	Improved antioxidant properties	Poor thermal stability Low chemically stability	(187)
Oil	l-ascorbic acid palmitic acid ester	Prevent lipid oxidation	Poor thermal stability Low chemically stability Heat labile	(188)

## 7. Encapsulation technologies and delivery systems

**Figure 8:**
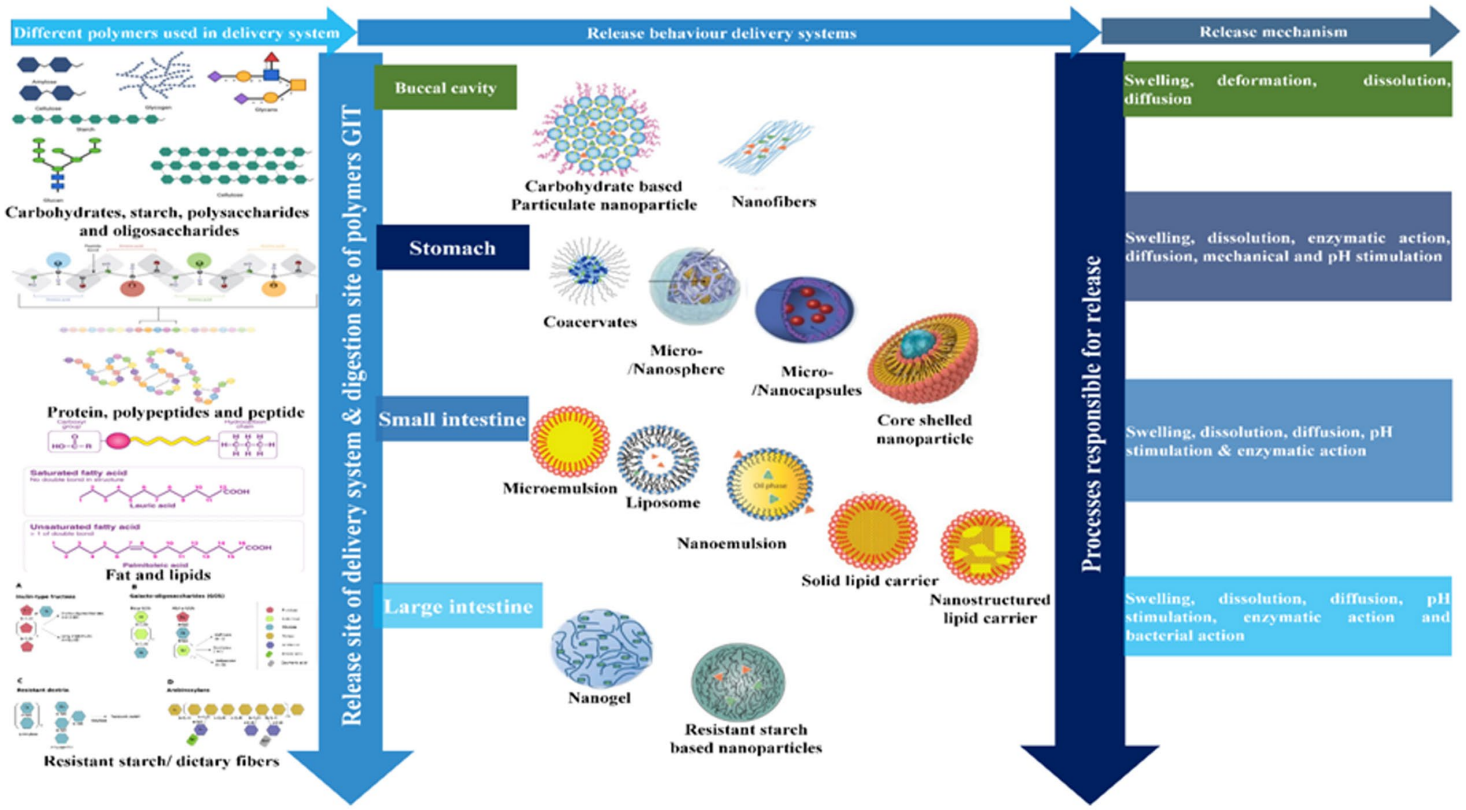
Fate of encapsulated delivery systems and their release mechanism and digestion site.

Encapsulation technologies are being used to improve the matrix compatibility, stability, and bioavailability of vitamins in fortified foods (15, 17, 171). A wide range of these technologies are available [Table 7 and Figure 8; (9, 11, 245, 246). At present, however, a universal encapsulation technology has not been developed that is applicable to all food products. Instead, they usually must be designed for the specific application, taking into account food matrix effects, and the nature of the processing, storage, and distribution conditions the food product will experience throughout its lifetime. Additionally, the knowledge of the performance of different vitamin C encapsulation technologies is still limited. For the sake of convenience, some of the most common encapsulation technologies developed for this purpose are classified into three groups based on the major ingredients used to fabricate them: polymer-, lipid-, and amphiphile-based systems (Supplementary Table S1). Nevertheless, some of these encapsulation technologies do combine two or more of these ingredients together.

**Table 7:** Encapsulation technique adopted for vitamin C encapsulation.

Encapsulation techniques	Wall material	Particle characterization				Potential application	References
		Particle size	Encapsulation efficiency	Release behaviors	Stability/Morphology/		
Spray drying	Casein	5.8 –14.8		Fast release	Stable at low pH/Irregular and porous	Food and infant formula	(208)
	Sodium alginate and chitosan, modified chitosan	3 μm	41.8–55.6%	Sustained release	Rough surface: Microparticle derived with chitosan Rough surface: Microparticle derived with modified chitosan	Pharmaceutical and food	(209)
	Chitosan and tripolyphosphate	4.1–7.3	58.3–68.7	Fast release	Spherical smooth surface microparticle	Pharmaceutical and food	(210)
	Chitosan	6.1–9.0	45.5–58.30	Fast release	Spherical smooth surface microparticle	Pharmaceutical	(211)
	Pea protein isolates, cowpea protein isolates	1.23–8.37			High vitamin C retention (65–69.30%)/Irregular shape	Food application	(212)
	Gum Arabic and modified starch	1087–1245 μm		Shown controlled release of AA during *invitro* digestion	Offered high vitamin C retention during storage period (9 weeks)	Pharmaceutical and food	(213)
	Taro starch	14.5–18.7 μm	20.9 ± 0.30%		High retention 80% after 6 weeks storage	Nutraceutical supplements	(214)
	Arabic gum	9.3	> 97		Microparticle offered 17% high retention than that of free vitamin C	Encapsulation of bioactive for bakery products	(215)
	Eudragit® RL, L and RS.		>95	Slow release		Pharmaceutical	(216)
	Maltodextrin and starch	4.75–7.6	100		High vitamin C retention (81–85%) after 60 days at room temperate Irregular and porous	Pharmaceuticals	(217)
	Maltodextrin and gum Arabic		>95		High retention after 300 days	Encapsulation of bioactive for bakery products	(218)
	Pea protein and sodium-carboxymethylcellulose	1.83–8.21	>84	Fast release	Pea protein microparticle: Quite irregular, shriveled and rough sodium-carboxymethylcellulose homogeneous and smooth	Food and Pharmaceuticals	(219)
	Starch, gum Arabic and gelatin	8.0–20.5	10.30		High vitamin C stability at ambient condition/Polyhedric microcapsules		(220)
	Sodium Alginate and Gum Arabic	2.88–14.09 μm	>90%		Spherical regular shape/Stable at higher temperature (188°C)	Nutraceutical supplements and food fortification	(221)
Spray chilling/spray cooling	Hydrogenated vegetable fat and stearic acid	31 2 μm	97.8		Microparticle have shown 13% high retention than that of free vitamin C	Suitable for bakery products	(222)
	Oleic acid (OA) and lauric acid (LA)	18–67 μm	89 − 98	Slow release in aqueous medium	Microparticle were present in agglomerates	Nutraceutical supplements and food fortification	(223)
	Palm oil and hydrogenated palm oil	98–181 μm	80.22 − 93.51	slow controlled released behavior	Crystalline microparticle	Nutraceutical supplements and food fortification	(224)
	Palm oil and hydrogenated palm oil	84.63 ± 1.20 μm			74.25–83.07% vitamin C retention after 45 days	Nutraceutical supplements and food fortification	(225)
Complex coacervation	Gum Arabic and gelatin	52–84	98	Controlled release under defined conditions	32–44% vitamin C retention after 34 days storage	Nutraceutical supplements and food fortification	(226)
Supercritical fluid (SC-CO_2_) assisted encapsulation	Vitamin E and liposomes	0.911	32.97		High emulsion stability under cold storage for 20 days	Nutraceutical supplements and food fortification	(227)
Microchannel emulsification	Soybean oil	15–18		High bioavailability	Narrow size distribution	Nutraceutical supplements and food fortification	(228)
Microfluidic technique	Chitosan and Na_2_CO_3_/palm fat	195-343	73.4–96.6		High Vitamin C retention (56–99%) at 4 °C High Vitamin C retention (46–98%) at 20 °C	fortified food products	(229)
Fluidized bed coating	Ethylcellulose/Polymethacrylate/waxy coating material	*>*315		Microparticle having Al-stearate showed the best release profile	Agglomeration of microparticle	Pharmaceutical	(230)
Liposome	Cholesterol, DL-α-tocopherol and phosphatidylcholine		53–55	Controlled release behavior	Multilamellar microparticles	Infant food formulations	(231)
	Milk-based phospholipids	1.0	10		High retention under cold condition/resistant to pH variation/ unilamellar microparticle	Food applications	(232)
	DL-*α*-tocopherol, egg phosphatidylcholine and cholesterol	0.2–1.0	59		Stable against pasteurization	Milk fortification	(233)
Melt extrusion	Maltodextrin	500–1000		Sustained release	High vitamin C retention (70%)/Crystalline	Bakery products	(234)
	Maltodextrin	500–1000	96	Sustained release	High retention/Large particle size	Food fortification	(235)
	Fructo-oligosaccharide	300–1000		Sustained release	Provide high stability to encapsulated vitamin C/crystalline	Fortification of low moisture containing foods	(11)
Melt dispersion	Carnauba wax	∼50	*<*100		Small size capsules/Porous microparticles	Food fortification	(236)
Emulsion solvent evaporation	Ethylcellulose				Tough and flexible microparticle/less porous microparticles	Food fortification	(236)
	Arabic gum and maltodextrin	55–107		Controlled release	Crystalline	Food formulations	(237)
Pickering emulsions	Modified cellulose and chitosan	620 nm	90.3	Controlled release	Susceptible to degradation/Pickering emulsion	Pharmaceuticals	(238)
Emulsions and coacervation	Gelatin and sodium caseinate system		65-97	Controlled release	Irregular and porous microparticles	Pharmaceutical/ nutraceutical	(239)
Spray coating	Polyacylglycerol monostearate		80.7–94.2	Slow release (9.2% after 12 d) in beverages	Improve retention oxidation and moisture	Beverage/ Milk fortification	(240)
	Medium-chain triacylglycerol	2-5	88.9–95.0	Higher degree of release	High protection against oxidation	Beverage/Milk fortification	(241)
Co-crystallization	Lactose and sucrose	2–30	*>*90		Low drug loading capacity/high stability/crystalline	Food fortification	(242)
Immobilization/dispersion	Sodium alginate and hydrated zinc oxide	359	Sustained release (∼90% release after 6 h)		Enhanced stability/gel like structure	Food fortification	(243)
Cross-Linking and Coacervation	Chitosan and alginate	2.6	Controlled release in GIT		Stable against pH/acidic condition	Pharmaceuticals	(244)

### 7.1. Polymer-based delivery systems

Researchers have exploited the scaffolding ability of natural or synthetic polymers to fabricate polymer-based delivery systems compatible with vitamin C encapsulation. This class of delivery system includes nanofibers, inclusion complexes, capsules, and particles (Table 8). These delivery systems can be assembled using a range of ingredients and fabrication methods, including simple mixing, electrostatic complexation, antisolvent precipitation, injection-gelation, spray drying, freeze drying, cold setting, ionic gelation, and electrospinning, which are discussed in detail elsewhere (279–282).

**Table 8:** Nanodelivery system adopted for vitamin C encapsulation.

Class of delivery system	Delivery system	Subclass of delivery system	Fabrication process	Wall materials	Particle characterization		Key outcomes	Reference
					Particles size	Encapsulation efficiency		
Amphiphilic based delivery systems	Liposome		Film evaporation and micro fluidization	Soy phosphatidylcholine	70 − 130	48–50	Enhanced vitamin C stability Reduced lipid oxidation, agglomeration and premature release of encapsulated vitamin C Improve physicochemical stability	(247)
			Extrusion	Soy phospholipid Krill	NA	<100	multilamellar liposome demonstrated high stability than unilamellar	(248)
			Dehydration/rehydration	Soybean phosphatidylcholine	100–150 nm	31.64–34.63	High stability under cold conditions after 49 days of storage	(249)
			Dehydration–rehydration	Soy phosphatidylcholine	140–220	38	High potential for food fortification	(250)
			micro fluidization	Soy phosphatidylcholine	∼100	∼62	Vitamin C stability can be enhanced addition of sucrose and applying freeze drying	(251)
			Film hydration-ultrasonication	Lecithin	373	42	Highly stable nanoparticle	(252)
			Hydration with extrusion	Hydrogenated soy phosphatidylcholine	<120	∼100	Boosted antitumor activity	(253)
	Micelles		Emulsification	poly(ε-caprolactone)-*b*-poly(*N,N*-diethylaminoethyl methacrylate)-ss-*b*-poly(2-methacryloyloxyethyl phosphorylcholine)	NA	NA	Offered surface charge conversion and fast drug release	(254)
Lipid based delivery systems	Nanostructured lipid carrier		High pressure homogenization	Witepsol®, Miglyol 812® TegoCare 450® Carbopol 940®	221	71.1	High stability under cold condition	(255)
			High pressure homogenization	Labrasol, Tristearin Phospholipid-90NG	268	87	Offer great drug target delivery	(256)
	Solid lipid carrier		High pressure homogenization	high pressure homogenization technique	228	67.6	High stability under cold condition	(255)
			Spray congealing	glycerol monostearate 90 Tween 80		74 - 84	Retained 75% of its initial vitamin C after 56 days of storage	(257)
	Microemulsion/Nanoemulsion		Emulsification	carboxymethyl cellulose, oleic acid as oil phase, Tween 20, propylene glycol	20-200 nm	NA	Offered high stability at various storage temperature (4°, 25° and 40 °C)	(258)
			Emulsification and titration	l-ascorbic acid, *β*-carotene, 1-pentano	NA	NA	Prevention of oxidation of β-carotene	(259)
			Spontaneous emulsification	Tween 20, tween 80, span 80, starch and virgin olive oil.	1,000 ± 68 nm	NA	Encapsulated vitamin C inhibited oxidation of olive oil	(260)
			Emulsification	Vitamin C, D-limonene, Tween20, Tween 80 and polyethylene glycol 400	55.65 ± 1.44–142.20 ± 7.82 nm	NA	Offer high stability at different storage temperature (25 and 40°C) after 1 month	(261)
			Emulsification	fish gelatin	97.45 ± 0.53	NA	Offer high antibacterial activity against bacterial film	(262)
	Molecular inclusion		Co-precipitation, kneading and freeze-drying	β-cyclodextrin	NA	NA	H NMR and UV-Vis, analysis	(263)
			Electro and physicochemical methods	*β*-cyclodextrin	NA	NA	FTIR, H NMR, UV-Vis, X-ray and DSC spectrum was performed	(264)
	Micro-/nanocapsules		Coacervations	soybean protein isolate (SPI)/pectin	16.24–24.12	78.80–91.62	Offer controlled release	(265)
			Coacervation	gelatin/sodium carboxymethyl cellulose	90–160	32.54–69.91	Offers good dispersibility and oral organoleptic attributes	(266)
			Coacervation	Gelatin and pectin	<10 μm	23.7–94.3	High release (68%) in the gastric fluid	(267)
			Coacervation	Gelatin and gum Arabic	7.7–12.4 μm	27.3–93.8	Offer high stability and release at defined pH conditions	(268)
			spray drying technique	Sodium alginate	NA	93.48	Vitamin C retained after 30 days of storages	(269)
			Coacervation	Gelatin and sodium caseinate	NA	8–99	Offer controlled release of encapsulated vitamin C	(239)
			Spray drying Solvent evaporation Melt dispersion method	Starch and -cyclodextrin	NA	NA	Delayed degradation of encapsulated vitamin C	(125)
			Spray drying Freeze drying	Arabic gum, stearic acid and hydrogenated vegetable fat	9.3–31.2 μm	97.8–100.8	Spray dried microcapsule has higher retention power than freeze drying microcapsules	(215)
			Complex coacervation and freeze drying	Corn oil and gelatin	26.59–81.91 ± 4.99	98	Improve vitamin C stability Offer controlled release under defined condition	(226)
		Micro-/nanospheres		Spray drying	Chitosan, tripolyphosphate	6.1–9.0 μm	45.05–58.30	Sustained release of encapsulated vitamin C	(211)			
	Solvent evaporation	Cellulose triacetate, ethylcellulose	NA	NA	Improved release at pH 7.4	(270)			
	Spray-drying	Eudragit® RL	NA	NA	Good particle size distribution and morphology	(216)
		Particular	Chitosan based nanoparticles	Ionic gelation	Chitosan Sodium tripolyphosphate	186–201	10–12	Improved stability against heat processing	(271)			
	Self-aggregation	chitosan	215.6 ± 18.1–288.2 ± 10.2 nm	55–67	Demonstrated resistance against gastric digestion	(272)			
	Chitosan	Ionic gelation	375–503	83–89	High vitamin C encapsulation Enhanced shelf life	(273)			
	N,N,N-trimethyl chitosan	Ionic gelation	∼530	N/A	Enhanced vitamin C stability	(274)			
	Chitosan	Ionic gelation	255.3 ± 22.9 nm	NA	Provide enhanced vitamin C stability under *in vitro* digestion	(275)			
	Chitosan Sodium tripolyphosphate	Ionic gelation	185	∼50	Controlled release	(276)		
	Starch nanoparticles	Potato starch	Ultrasonication	N/A	42-80	High stability against heat processing	(277)	
	Nanofiber		Polyvinyl alcohol	Electrospinning process	50	NA	Porous in nature Fast release of encapsulated vitamin C	(278)

#### 7.1.1. Microfibers/nanofibers

Microfibers or nanofibers can be prepared using one or more natural or synthetic polymers (283, 284). Typically, a microfiber has a diameter above a few hundred nanometers whereas a nanofiber had a diameter below this value, but there is no clear cut off. A range of fabrication techniques have been used to create polymer fibers including centrifugal spinning and electrospinning, with the latter being most explored for encapsulation applications (285–291). The functional properties of electrospun fibers can be controlled by varying their composition, dimensions, and surface properties, which is useful for controlling the dispersibility, stability, and release behaviors of encapsulated vitamins (292–296). The use of polymer fibers for vitamin C encapsulation is still in its infancy, with few published studies in this area. One group developed vitamin C-loaded polyvinyl alcohol/β–cyclodextrin nanofibers suitable for applications in cosmetics, personal-care products, and topical drug delivery (297). Fish oil/gelatin nanofibers produced by electrospinning have also been used to encapsulate vitamin C (298).

#### 7.1.2. Molecular inclusion complexes

Generally, polymeric molecular inclusion complexes are produced from polymers and other host molecules capable of binding guest molecules. Cyclodextrins are the most widely used substances to encapsulate bioactive compounds. They have a cavity that can accommodate guest molecules due to the formation of a helix by α(1,4)-linked glucose chains (299). Vitamin C can be incorporated into this cavity (300). Molecule inclusion complexes can be prepared using various methods, including solvent evaporation, isoelectric precipitation, mixing, and freeze-drying (301–306). The structural and physicochemical properties of vitamin C-loaded β-cyclodextrin molecular inclusion complexes formulated using different approaches (co-precipitation, kneading, and freeze-drying) have been characterized (263). Other researchers have also reported that β-cyclodextrin can be used to encapsulate vitamin C (264).

#### 7.1.3. Polymer capsules and particles

Polymer capsules consist of polymeric shells surrounding fluid cores, whereas the whole of polymer particles consist of a polymer network. For the sake of clarity, these will both be referred to as polymer particles, unless otherwise stated. Polymer particles may be assembled from synthetic and/or natural polymers. In the food industry, proteins and polysaccharides are typically used for this purpose. Typically, microcapsules/microparticles have diameters more than a few hundred nanometers, whereas nanocapsules/nanoparticles have smaller dimensions. Polymer particles can be formed by various methods, including injection-gelation, coacervation, spray-drying, freeze-drying, solvent displacement, templating, and molding (307–312). A few studies have demonstrated the potential of polymer particles for vitamin C encapsulation. For instance, vitamin C was encapsulated in gelatin-based microparticles prepared using the coacervation method to improve its stability and control its release (226). Similarly, casein hydrolysate/soy protein/pectin particles have been used to improve vitamin C stability (265). Furthermore, polymer microparticles were fabricated using coacervation for co-encapsulation of vitamin C and quercetin (262). Some researchers have investigated the effects of different fabrication methods on the release of vitamin C from polymer microparticles (236). The retention and release behavior of vitamin C in gelatin-caseinate microparticles has also been studied (239). Studies have shown that vitamin C is released from gelatin-pectin microparticles under simulated gastrointestinal conditions (267). Alginate-based microparticles have been shown to retain vitamin C throughout 30 days of storage (269). Some researchers have examined the impact of introducing encapsulated vitamin C into food products. For instance vitamin C-loaded microparticles have been incorporated into bakery products (215). In this case, encapsulation was shown to increase the stability of the vitamin.

Several different kinds of food-grade polymer particles are summarized in Table 8. Chitosan is often used to assemble these systems because it is a cationic polysaccharide that can bind anionic vitamin C. For instance, vitamin C-chitosan nanoparticles have been shown to improve the bioavailability of the vitamin (276). In another study, researchers developed vitamin C-loaded nanoparticles using the ionic gelation method to improve vitamin C stability against heat processing (271). Encapsulation of vitamin C in chitosan-based nanoparticles has also been shown to improve it stability during storage (273) and to prolong its release under gastric fluid conditions (275). Vitamin C was also encapsulated in starch-derived nanoparticles to improve its stability and bioavailability (277). Typically, the composition, dimensions, structure, surface properties, and pore size of polymer particles must be manipulated to control the retention, release, and stability of vitamin C.

### 7.2. Amphiphile-based delivery systems

Amphiphilic-based delivery systems are typically assembled from amphiphilic ingredients, such as phospholipids and surfactants. These amphiphilic substances tend to assemble into a colloidal structure like micelles, microemulsions, or liposomes due to the hydrophobic effect.

#### 7.2.1. Liposomes

Liposomes typically consist of phospholipid bilayers organized into one or more concentric shells. As a result, they have both hydrophilic domains (polar head groups and inner core) and lipophilic domains (non-polar tail groups). A variety of preparation methods have been developed to fabricate liposomes (313–317). Liposomes with diameters below a few hundred nanometers are often referred to as nanoliposomes. Several studies have shown that vitamin C can be encapsulated in liposomes (231, 233). For instance, researchers have shown that encapsulation of vitamin C in liposomes assembled from phosphatidylcholine, tocopherol, and cholesterol could improve its stability (231). Similarly, loading vitamin C into liposomes assembled from soybean phosphatidylcholine was shown to improve its resistance to oxidation and to premature release during digestion (247). Vitamin C has been co-encapsulated with vitamin A and methionine in liposomes (248). Vitamin C has been loaded into liposomes fabricated using a film hydration-sonication technique, which improved its stability (252). Some researchers have reported that vitamin C-loaded liposomes can be incorporated into milk products (233).

#### 7.2.2. Micelles and microemulsions

Conventional micelles and oil-in-water microemulsions consists of small colloidal particles assembled from surfactants, where the non-polar tails are the interior, and the polar heads are exposed to the surrounding water (318–320). Conversely, in reverse micelles and water-in-oil (W/O) microemulsions the polar heads are in the interior and the non-polar tails are exposed to the surrounding oil. These kinds of association colloids are thermodynamically stable systems under specific compositional and environmental conditions (321). Consequently, they can often be formed by simply mixing the different components together. The particle size of these systems is typically very low (<50 nm), which means that are optically transparent and highly resistant to gravitational separation (322). A variety of fabrication methods are available for encapsulating bioactive substances within association colloids, including solvent evaporation and spontaneous emulsification (319, 322–327). It is often difficult to encapsulate and retain vitamin C into micelles and oil in water (O/W) microemulsions because of its hydrophilic nature. However, it can be trapped within the internal water domain of reverse micelles or W/O microemulsions when the continuous phase is oil. Only a few studies have so far been conducted on the use of association colloids for vitamin C encapsulation and delivery. For instance, vitamin C has been loaded into micelles assembled from modified-phosphorylcholine and used as an antitumor drug delivery system (254). Researchers have fabricated microemulsions from carboxymethyl cellulose, oleic acid, Tween 20, and propylene glycol and observed them to be highly stable at different storage temperatures (4°, 25°, and 40°C) (258). The researchers also investigated the influence of surfactant/co-surfactant and hydrophilic-lipophilic balance on vitamin C-loaded microemulsions (261). In general, this kind of delivery system is likely to be most useful for applications where the vitamin C needs to be trapped within an oil phase, then reverse micelles or W/O microemulsions can be used.

### 7.3. Lipid-based delivery systems

This group of delivery systems includes colloidal dispersions primarily assembled from edible fats and oils, including emulsions, solid lipid nanoparticles, and nanostructured lipid carriers (Table 8).

#### 7.3.1. Emulsions

Emulsions are thermodynamically unstable colloidal dispersions because of the positive free energy associated with the oil-water interface. Emulsions with droplets below a few hundred nanometers are often referred to as nanoemulsions. A range of fabrication methods has been developed to form emulsions, including mechanical approaches (like microfluidization, homogenization, and sonication methods) and physicochemical approaches (like phase inversion and spontaneous emulsification methods) (328–330). Emulsions can be classified as oil-in-water or water-in-oil types depending on whether the oil phase makes up the droplets or the surrounding medium, respectively. O/W emulsions are rarely used to encapsulate vitamin C because it is hydrophilic and therefore tends to be soluble in the external aqueous phase, rather than inside the oil droplets. Researchers have encapsulated vitamin C within the internal aqueous phase of W/O/W multiple emulsions, but they did not measure its retention or stability over time (331). In another study, the same authors showed that the vitamin C was rapidly released from these emulsions, which can be attributed to the fact that it has some solubility in oil and can therefore diffuse out of the W/O droplets into the surrounding water (239). Emulsions therefore appear to have limited application for the encapsulation of vitamin C.

#### 7.3.2. Solid lipid nanoparticles and nanostructured lipid carriers

This type of colloidal delivery system is like an emulsion, but the lipid droplets are fully or partially crystalline. Typically, an oil-in-water emulsion is formed at a temperature above the melting point of the fat phase, and then the system is cooled to promote crystallization and form solid lipid nanoparticles (SLNs) or nanostructured lipid carriers (NLCs) (332–335). In SLNs, the lipid phase is completely crystalline, whereas in NLCs it is only partly crystalline. The advantages of using these kinds of delivery systems are that the solid nature of the lipid phase can slow down molecular diffusion processes, which can improve the retention and stability of encapsulated substances. SLNs and NLCs are typically used to encapsulate lipophilic bioactive substances but some researchers have examined their application to vitamin C. For instance, vitamin C-loaded SLNs have been prepared using a hot homogenization method (255). Another study reported that vitamin C was retained in SLNs at a relatively high level (>75%) after 56 days of storage (257). High-pressure homogenization has been used to produce vitamin C-loaded NLCs, which was shown to prolong the release of the vitamin (256). Nevertheless, further research is required in this area. Like emulsions, it may be difficult to trap and retain the hydrophilic vitamin C molecules within the hydrophobic interior of the particles in SLNs and NLCs.

## 8. Fate of vitamin C loaded delivery systems in gastrointestinal tract

It is often important to design food-grade delivery systems that can increase the bioavailability of nutrients and/or control the region they are released and absorbed in the gastrointestinal tract (9, 13–17, 67). This can often be achieved by controlling the compositions, sizes, structures, physical states, aggregation states, and interfacial properties of the colloidal particles they contain. Most research studies on vitamin C fortification using delivery systems have focused on the following aspects: morphological characterization, degree of stability enhancement, release kinetics, compatibility with food matrices, stability in food matrices, and impact on food properties (9). Some studies have also examined the bioaccessibility (*in vitro* models) or bioavailability (*in vivo* models) of encapsulated vitamin C.

It is important that any encapsulated vitamin C is released within the gastrointestinal tract in an active form that can be absorbed by the enterocytes. The hydrophilic nature of vitamin C means that it is usually highly soluble in gastrointestinal fluids, which ensures it has a high bioaccessibility (67). However, it may chemically degrade within the gastrointestinal environment, which can be inhibited using well-designed delivery systems. Nevertheless, there is still a need for a systematic comparison of the efficacy of different kinds of delivery systems for improving the bioavailability of vitamin C in different food matrices. A schematic diagram of the fate of vitamin C-loaded delivery systems in the human gastrointestinal tract is shown in Figure 8: (i) the delivery system should initially contain a sufficiently high concentration of the vitamin to have a biological effect; (ii) the delivery system should retain and protect the vitamin in the mouth and stomach; (iii) the delivery system should release the vitamin in the small intestine where absorption normally occurs; (iv) the delivery system might be designed to protect the vitamin and promote its absorption in the small intestine; (v) the delivery system itself should be safe for application within foods. Clearly, further studies are needed in this area.

## 9. Safety compliance and risks of vitamin C delivery systems

It is important that any vitamin C delivery systems are safe for human consumption and do not have any unforeseen adverse health effects (336, 337). Synthetic polymers or surfactants may have some undesirable health impacts and therefore natural alternatives may be better (338). Similarly, the use of organic solvents, alcohols, or synthetic chemicals during the production of the delivery systems should be avoided, or they should be completely removed prior to sale, to reduce health risks (338). In general, the impact of their short- and long-term effects on human health should be assessed (338). The Food and Drug Administration (FDA) in the United States has released guidelines regarding the incorporation of nanoparticles in foods (339). The European Food Safety Authority (EFSA) in the European Union has developed regulations on the utilization of nanomaterials as delivery systems in foods (340). Methods to perform risk assessments of nanomaterials applied in foods have been given (341).

## 10. Conclusion

In many countries, the general population consumes enough fruits and vegetables to have sufficient levels of vitamin C in their diets. However, there are some populations that do suffer from vitamin C deficiencies, which lead to debilitating diseases like scurvy. Moreover, vitamin C may act as a nutraceutical ingredient that can exhibit a range of other beneficial health effects, especially due to its antioxidant activity. The biological activity of vitamin C in many foods and beverages is limited because of its tendency to chemical degrade. Consequently, there is interest in improving the chemical stability and bioavailability of this bioactive substance using encapsulation technologies. There have been many studies on the use of colloidal delivery systems to encapsulate, protect, and release hydrophobic vitamins (like vitamins A, D, and E) but to far fewer on their application to hydrophilic vitamins (like vitamin C). There appears to be a range of colloidal delivery systems available that can be used for this purpose, especially those that have hydrophilic domains inside the particles (like polymer particles, W/O/W emulsions, and liposomes) but further work is needed to establish their relative merits and limitations. Moreover, research is required to establish whether they can be affordably produced at sufficiently high quantities for commercial applications, and whether they are robust enough and effective under real life situations.

## Author contributions

VM: conceptualization, methodology, writing – original draft, writing – review and editing, and data curation. AS: methodology, writing – review and editing, writing – original draft, and conceptualization. DM: project administration, supervision, visualization, writing – review and editing. RS: data curation, formal analysis, and visualization. KB: investigation, data curation, methodology, and formal analysis. TR: methodology, formal analysis, and resources. JL: funding acquisition, resources, validation, and review. ES: funding acquisition, resources, and validation. All authors contributed to the article and approved the submitted version.

## Funding

This work was supported by the Priority Research Centres Program through the National Research Foundation of Korea (NRF), funded by the Ministry of Education (2014R1A6A1031189) and by an NRF grant funded by the Korean government (MSIT) (Grant No. 2021R1A2C1008368).

## Conflict of interest

VM was employed by the company PerkinElmer.

The remaining authors declare that the research was conducted in the absence of any commercial or financial relationships that could be construed as a potential conflict of interest.

## Publisher’s note

All claims expressed in this article are solely those of the authors and do not necessarily represent those of their affiliated organizations, or those of the publisher, the editors and the reviewers. Any product that may be evaluated in this article, or claim that may be made by its manufacturer, is not guaranteed or endorsed by the publisher.
