# Supplementary material for: Vitamin C fortification: need and recent trends in encapsulation technologies
Source: Front Nutr. 2023 Sep 7;10:1229243. doi: 10.3389/fnut.2023.1229243 (PMC10517877; doi:10.3389/fnut.2023.1229243)
Supplement: Supplementary file 1 [file Table_1.DOCX]

**Table S1:** Nanodelivery system adopted for vitamin C encapsulation

| **Class of delivery system** | **Delivery system** | **Subclass of delivery system** | **Fabrication process** | **Wall materials** | **Particle characterization** | | **Key outcomes** | **Reference** |
| --- | --- | --- | --- | --- | --- | --- | --- | --- |
|  |  |  |  |  | **Particles size** | **Encapsulation efficiency** |  |  |
| Amphiphilic based delivery systems | Liposome | | Film evaporation and micro fluidization | Soy phosphatidylcholine | 70 − 130 | 48-50 | Enhanced vitamin C stability  Reduced lipid oxidation, agglomeration and premature release of encapsulated vitamin C  Improve physicochemical stability | (Zhou et al., 2014) |
|  |  | | Extrusion | Soy phospholipid  Krill | NA | <100 | multilamellar liposome demonstrated high stability than unilamellar | (Monroig, Navarro, Amat, & Hontoria, 2007) |
|  |  | | Dehydration/rehydration | Soybean phosphatidylcholine | 100–150 nm | 31.64 -34.63 | High stability under cold conditions after 49 days of storage | (Jiao, Wang, Yin, & Xia, 2019) |
|  |  | | Dehydration–rehydration | Soy phosphatidylcholine | 140-220 | 38 | High potential for food fortification | (Marsanasco, Márquez, Wagner, del V. Alonso, &Chiaramoni, 2011) |
|  |  | | micro fluidization | Soy phosphatidylcholine | ∼100 | ∼62 | Vitamin C stability can be enhanced addition of sucrose and applying freeze drying | (Shuibing Yang et al., 2013) |
|  |  | | Film hydration-ultrasonication | Lecithin | 373 | 42 | Highly stable nanoparticle | (SB Yang et al., 2010) |
|  |  | | Hydration with extrusion | Hydrogenated soy phosphatidylcholine | <120 | ∼100 | Boosted antitumor activity | (Lipka et al., 2013) |
|  | Micelles | | Emulsification | poly(ε-caprolactone)-b-poly(N,N-diethylaminoethyl methacrylate)-ss-b-poly(2-methacryloyloxyethyl phosphorylcholine) | NA | NA | Offered surface charge conversion and fast drug release | (Wu, Chen, Gan, Chen, & Luo, 2020) |
| Lipid based delivery systems | Nanostructured lipid carrier | | High pressure homogenization | Witepsol®, Miglyol 812® TegoCare 450® Carbopol 940® | 221 | 71.1 | High stability under cold condition | (Üner, Wissing, Yener, & Müller, 2005) |
|  |  | | High pressure homogenization | Labrasol, Tristearin Phospholipid-90NG | 268 | 87 | Offer great drug target delivery | (A. Jain et al., 2016) |
|  | Solid lipid carrier | | High pressure homogenization | high pressure homogenization technique | 228 | 67.6 | High stability under cold condition | (Üner et al., 2005) |
|  |  | | Spray congealing | glycerol monostearate 90  Tween 80 |  | 74 - 84 | Retained 75% of its initial vitamin C after 56 days of storage | (Matos-Jr, Di Sabatino, Passerini, Favaro-Trindade, & Albertini, 2015) |
|  | Microemulsion/Nanoemulsion | | Emulsification | carboxymethyl cellulose, oleic acid as oil phase, Tween 20, propylene glycol | 20-200 nm | NA | Offered high stability at various storage temperature (4°, 25° and 40 °C) | (Vitamin, 2015) |
|  |  | | Emulsification and titration | L‐ascorbic acid, β‐carotene, 1‐pentano | NA | NA | Prevention of oxidation of β‐carotene | (Szymula, 2004) |
|  |  | | spontaneous emulsification | Tween 20, tween 80, span 80, starch and virgin olive oil. | 1,000 ± 68 nm | NA | Encapsulated vitamin C inhibited oxidation of olive oil | (Osanloo, Jamali, & Nematollahi, 2021) |
|  |  | | Emulsification | Vitamin C, D-limonene, Tween20, Tween 80 and polyethylene glycol 400 | 55.65 ± 1.44- 142.20 ± 7.82 nm | NA | Offer high stability at different storage temperature (25 and 40°C) after 1 month | (Ramli, Chyi, Zainuddin, Mokhtar, & Abdul Rahman, 2019) |
|  |  | | Emulsification | fish gelatin | 97.45 ± 0.53 | NA | Offer high antibacterial activity against bacterial film | (M. Ji et al., 2021) |
|  | Molecular inclusion | | co-precipitation, kneading and freeze-drying | β-cyclodextrin | NA | NA | H NMR and UV-Vis, analysis | (Bratu, Muresan-Pop, Kacso, & Fărcaş, 2009) |
|  |  | | Electro and physicochemical methods | β-cyclodextrin | NA | NA | FTIR, H NMR, UV-Vis, X-ray and DSC spectrum was performed | (Saha, Roy, Roy, & Roy, 2016) |
|  | Micro-/nanocapsules | | Coacervations | soybean protein isolate (SPI)/pectin | 16.24 - 24.12 | 78.80- 91.62 | Offer controlled release | (Mendanha et al., 2009) |
|  |  | | Coacervation | gelatin/sodium carboxymethyl cellulose | 90-160 | 32.54-69.91 | Offers good dispersibility and oral organoleptic attributes | (R. Ji et al., 2021) |
|  |  | | Coacervation | Gelatin and pectin | <10µm | 23.7 to 94.3 | High release (68%) in the gastric fluid | (da Cruz, Perussello, & Masson, 2018) |
|  |  | | Coacervation | Gelatin and gum arabic | 7.7-12.4μm | 27.3-93.8 | Offer high stability and release at defined pH conditions | (Rodrigues da Cruz, Andreotti Dagostin, Perussello, & Masson, 2019) |
|  |  | | spray drying technique | Sodium alginate | NA | 93.48 | Vitamin C retained after 30 days of storages | (Marcela, Lucía, Esther, & Elena, 2016) |
|  |  | | Coacervation | Gelatin and sodium caseinate | NA | 8-99 | Offer controlled release of encapsulated vitamin C | (Fraj et al., 2021) |
|  |  | | Spray drying  Solvent evaporation  Melt dispersion method | Starch and ­ -cyclodextrin | NA | NA | Delayed degradation of encapsulated vitamin C | (M. S. Uddin, 2001) |
|  |  | | Spray drying  Freeze drying | arabic gum, stearic acid and hydrogenated vegetable fat | 9.3- 31.2 μm | 97.8-100.8 | Spray dried microcapsule has higher retention power than freeze drying microcapsules | (Alvim et al., 2016) |
|  |  | | Complex coacervation and freeze drying | corn oil and gelatin | 26.59- 81.91 ± 4.99 | 98 | Improve vitamin C stability  Offer controlled release under defined condition | (T. A. Comunian et al., 2013) |
|  | Micro-/nanospheres | | spray drying | Chitosan, tripolyphosphate | 6.1–9.0 µm | 45.05–58.30 | Sustained release of encapsulated vitamin C | (K. G. H. Desai & Park, 2005) |
|  |  | | Solvent evaporation | cellulose triacetate, ethylcellulose | NA | NA | Improved release at pH 7.4 | (Khaldia, Lamia, Yasmina, & Lahcene, 2020) |
|  |  | | Spray-drying | Eudragit^®^ RL | NA | NA | good particle size distribution and morphology | (Esposito et al., 2002) |
|  | Particular | Chitosan based nanoparticles | Ionic gelation | Chitosan  Sodium tripolyphosphate | 186-201 | 10-12 | Improved stability against heat processing | (Jang & Lee, 2008) |
|  |  |  | Self-aggregation | chitosan | 215.6 ± 18.1- 288.2 ± 10.2nm | 55-67 | Demonstrated resistance against gastric digestion | (Cho, Kim, & Park, 2012) |
|  |  |  | Chitosan | Ionic gelation | 375–503 | 83-89 | High vitamin C encapsulation  Enhanced shelf life | (Aresta et al., 2013) |
|  |  |  | N,N,N-trimethyl chitosan | Ionic gelation | ∼530 | N/A | Enhanced vitamin C stability | (de Britto, de Moura, Aouada, Mattoso, & Assis, 2012) |
|  |  |  | Chitosan | Ionic gelation | 255.3 ± 22.9nm | NA | Provide enhanced vitamin C stability under in vitro digestion | (Dudhani & Kosaraju, 2010) |
|  |  |  | Chitosan  Sodium tripolyphosphate | Ionic gelation | 185 | ∼50 | Controlled release | (Alishahi et al., 2011) |
|  |  | Starch nanoparticles | Potato starch | Ultrasonication | N/A | 42-80 | High stability against heat processing | (Shabana et al., 2019) |
|  | Nanofiber |  | Polyvinyl alcohol | Electrospinning process | 50 | NA | Porous in nature  Fast release of encapsulated vitamin C | (Tehrani & Amiri, 2022) |
